# Novel use of Absorbable Modified Polymer (AMP®); EndoClot™ as an adjunct in the management of bleeding from the liver bed during laparoscopic cholecystectomy

**DOI:** 10.1186/s40064-015-1031-6

**Published:** 2015-06-11

**Authors:** Pramodh Chitral Chandrasinghe, Asantha De Silva, Kemal Ismail Deen

**Affiliations:** Department of Surgery, Faculty of Medicine, University of Kelaniya, Kelaniya, Sri Lanka; Department of Obstetrics and Gynaecology, Faculty of Medicine, University of Kelaniya, Kelaniya, Sri Lanka

**Keywords:** Absorbable modified polymer, Hemorrhage, Laparoscopy

## Abstract

Absorbable modified polymer (AMP) is a novel local haemostatic agent derived from a natural polysaccharide. Its safety and efficacy has been evaluated in upper and lower gastrointestinal bleeding without reported side effects. We report the safe use of AMP as an adjunct in the management of serious bleeding during laparoscopic cholecystectomy.

## Background

Serious intra-operative bleeding during laparoscopic cholecystectomy is rare with an incidence of less than 2% (Tuveri and Tuveri 2007; Marakis et al. 2007; Vagenas et al. 2006). The incidence is high in patients with liver disease, specifically, in cirrhosis with portal hypertension (Kaushik 2010). Absorbable Modified Polymer (AMP®) is a polysaccharide which is sprayed on to a bleeding surface and acts by absorbing fluid and increasing the concentration of platelets, red blood cells and clotting factors at the site. Although short case series of using AMP in gastrointestinal bleeding are available (Halkerston et al. 2013; Patel et al. 2014) this is the first report of its intra-peritoneal use.

### Case presentation

**Figure 1:**
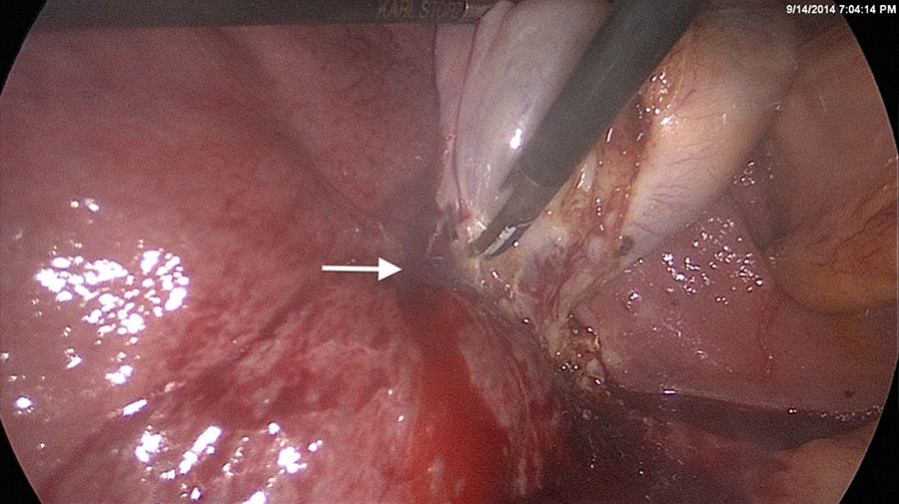
The site of liver bed bleeding (*arrow*) due to detachment of the peritoneal attachment of gall bladder.

**Figure 2:**
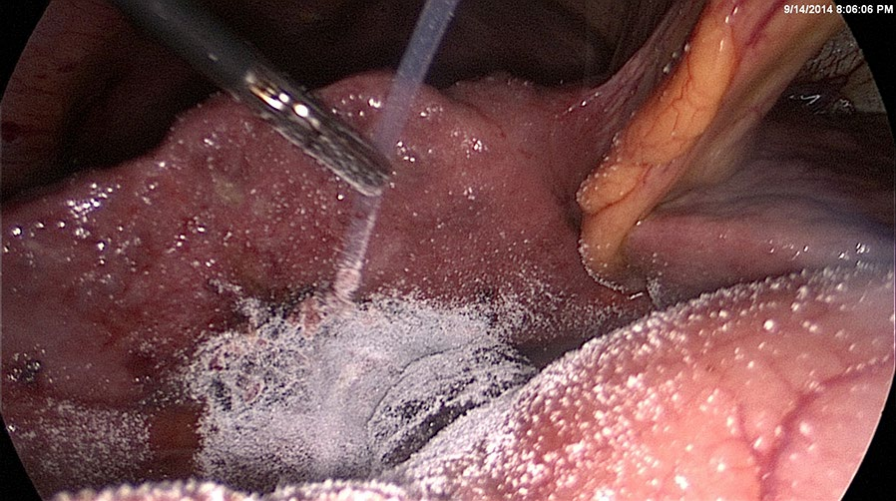
AMP EndoClot™ being applied to the bleeding gall bladder bed via the plastic catheter guided by a grasper.

A 36 year old male with child’s C cirrhosis presented with a history of repeated cholangitis resulting in encephalopathy. His clinical presentation was supported by further derangement of his liver enzyme status, serum bilirubin, and elevation in serum alkaline phosphatase levels. Trans-abdominal ultrasonic imaging revealed a dilated common bile duct with a calculus at its lower end and multiple calculi in the gallbladder. He was treated with intravenous antibiotics which resulted in improvement in his clinical status and part reversal of his biochemical parameters. A subsequent magnetic resonance cholangio-pancreatogram (MRCP) revealed that the calculi had spontaneously passed through the common bile duct, obviating the requirement for endoscopic extraction. Despite clinical improvement, his international normalized ratio (INR) continued to remain high at 2.5. To prevent further compromization of his liver function, a multi-disciplinary decision was made to undertake laparoscopic cholecystectomy. Following peri-operative transfusion of fresh frozen plasma (15 ml/kg) and parenteral vitamin K, his INR improved to 2; the platelet count was 1,300,000/mm^3^. Under general anesthesia, we undertook standard 4-port laparoscopic cholecystectomy as described previously (Liyanage et al. 2006). In brief, pneumoperitoneum was achieved through an umbilical port, to maintain an intraperitoneal carbon dioxide pressure of 12 mmHg. The liver appeared shrunken with an irregular surface. There were no significant varices around the Calot’s triangle. As the gallbladder was gently retracted cephalad with an atraumatic grasper the peritoneum over the posterior surface of liver became detached which resulted in a minor bleed (Figure 1). The hemorrhage was arrested temporarily with local pressure. Simultaneously, the cystic duct and artery in Calot’s triangle were identified, clipped and divided, and the gallbladder was separated from the liver bed with use of an ultrasonic dissector. At the end of gallbladder removal, which took approximately 35 min, bleeding that had commenced at the inferior edge of the gall bladder attachment to the liver bed, was seen from the entire raw surface of the liver. High wattage electro-cautery of the bared liver surface combined with several applications of activated cellulose meshes failed to achieve satisfactory hemostasis. We used 2 g of absorbable modified polymer Endoclot™ (EndoClot Plus Inc, Santa Clara, CA, USA) which was sprayed over the surface of the bared liver to achieve hemostasis. The endoclot powder spray was delivered intra-peritoneally through a plastic catheter, provided by the manufacturer, introduced via a 5 mm reducer tube through the epigastric 10 mm port. This step is key, as passage of the catheter without the tube would have resulted in kinking of the catheter at the site of the port valve. The free end of the delivery catheter was connected to an air compressor pump device and the entire assembly activated at the pump switch as per manufacturer’s instructions. The tip of the catheter was aimed at the site of bleeding using an atraumatic grasper (Figure 2). Thus, hemostasis was achieved rapidly and confirmed by laparoscopic observation for 10 min. The blood in the peritoneal cavity was sucked out and the port sites were closed after evacuation of pneumo-peritoneum. The patient was discharged from hospital after having made satisfactory recovery, without further blood loss.

## Discussion

AMP is a polysaccharide derived from natural sources (Endoclotplus 2014). The substance has no reaction with components of human blood but promotes clotting through a dehydrating mechanism increasing the concentration of clotting factors and cells at the site. The residual particles are lysed by amylase and glucoamylase and is completely cleared from the site within few hours to days (Endoclotplus 2014). Halkerston et al. (2013) reported in a short case series of six patients treated with AMP as an adjunct in the management of upper gastrointestinal bleeding and colonic bleeding following endoscopic mucosal resection (EMR). Patel et al. 2014 recently reported the safety and efficacy of AMP in 18 patients with a 89% (n = 16) success in achieving hemostasis. In their case series the upper intestinal bleeds (n = 16) were initially managed with adrenaline injections, clipping and diathermy whereas for the lower intestinal bleeds (n = 3), all following EMR, argon plasma coagulation was also utilized. In two patients who developed delayed hemorrhage, despite initial response to AMP, the authors found a gastro intestinal stromal tumour (1) and a Dieulafoy lesion (2), suggesting that use of Endoclot™ must be restricted to a facilitatory role and should not replace conventional attempts to achieve hemostasis. Our patient was a high-risk candidate for intraoperative bleeding due to existing liver failure. As bleeding occurred from the surface of the liver application of clips was not technically possible. Although AMP Endoclot™ has not been previously used at an intra-peritoneal site, there is evidence for safety of its use on raw surfaces as reported in EMR of colonic polyps (Halkerston et al. 2013; Patel et al. 2014). None of the case series in the literature has reported side effects with AMP usage.
